# The Internet Use for Health Information Seeking among Ghanaian University Students: A Cross-Sectional Study

**DOI:** 10.1155/2017/1756473

**Published:** 2017-10-31

**Authors:** Benedict Osei Asibey, Seth Agyemang, Augustina Boakye Dankwah

**Affiliations:** ^1^Department of Geography and Rural Development, Faculty of Humanities and Social Sciences, Kwame Nkrumah University of Science and Technology, Kumasi, Ghana; ^2^Department of Geography and Resource Development, Faculty of Social Sciences, University of Ghana, Legon, Accra, Ghana

## Abstract

The aim of the study was to investigate university students' use of the Internet for health purpose in the Ghanaian context. The study employed a quantitative cross-sectional design. A total of 650 out of 740 students selected from 3 different universities participated, giving a response rate of 87.7% (650/740). Data were obtained using questionnaires and frequency and percentages were used to analyze data. The results show that university students are active users of the Internet as 78.3% (509/650) used Internet daily and 67.7% (440/650) use Internet for health purposes, for reasons including availability and ease of accessing information, privacy, confidentiality, and affordability. Use of Internet was constrained by unreliable and slow connection, high cost of Internet, and unreliable power supply. Also, 72.4% (315/435) used the online health information obtained as a basis for lifestyle change and only 39.5% (170/430) consulted health professionals after obtaining online information. The study concludes that students use Internet to seek online health support. The use of Internet to communicate with young people in relation to their health must therefore be explored. There is the need to be aware of online safety issues for young adults, including the need to provide information on privacy options.

## 1. Introduction

The Internet is a global network “information superhighway” that enables computers and other communication gadgets to communicate directly and transparently [1]. Internet is defined as a global broadcasting capability, a system for information broadcasting, and a means for interaction and collaboration between individuals and their communication devices irrespective of their geographical location [2].

The Internet, given its availability, affordability, and versatility, is used for different purposes, including increasing health-related purposes. The Internet is a very popular and important source of health information for both healthcare professionals and patients, as it offers the formal access to high quality, huge volume, current, and relevant healthcare information [3]. Thus, it has become increasingly common for people to locate health information and support with the use of Internet worldwide [4, 5]. For instance, the Pew Internet and American Life Project in 2000 and 2009, respectively, reported that 55% and 80% of adults in the United States of America who had access to Internet used it to obtain medical or health information and support [4, 6, 7]. In Europe, the Health Service Executive in 2007 reported that 18% of Irish people used Internet for health information [8, 9].

In spite of the benefits of using Internet for health purpose, the existing literatures have mainly focused on developed countries, with little research work done in developing countries, particularly Africa. In order to bridge this knowledge gap there is the need to examine the use of the Internet for health purposes among the youth in sub-Saharan Africa. This study focused on university students in Ghana.

## 2. Background to Ghana's Healthcare Services

Access to basic healthcare services in Ghana still remains a huge challenge, and average life expectancy is about 10 years below the global average [10]. Preventable diseases such cholera and malaria continue to exert a heavy toll on the population. Other issues such as HIV AIDS, tuberculosis, and infant, child, and maternal mortality are still a force to reckon with, in spite of the giant strides made so far. Health professionals and healthcare services are being overwhelmed by the increasingly huge demand for healthcare. Though gradually improving, Ghana's doctor-to-patient ratio is still alarming. It was 1 : 10,341 in 2010, 1 : 10,170 in 2013, and stood at 1 : 9,043 in 2014. This is below the WHO standard of 1 : 600. The nurse-to-population ratios of 1 : 1,516 in 2010, 1 : 1,084 in 2013, and 1 : 959 in 2014 still fall below the WHO standard. The picture painted is that of inadequate number of health professionals against a population of 25.90 million [10]. The challenge of access is compounded by inadequate funds to finance health operations, poor and inadequate health infrastructure, and shortage of qualified health professionals.

It becomes imperative therefore to find alternative means of seeking healthcare that meet the minimum qualifications. One emerging innovation is to make health information accessible to all on a platform which can be reached by all irrespective of location and socioeconomic background, which is the Internet. Previous studies in Ghana have revealed that several Ghanaians use the Internet for different purposes [11–15]. The liberalization of the telecommunications sector allowing the market to be inundated with so many companies offering attractive packages and bonuses has certainly contributed to this boost. Statistics from the National Communication Authority (NCA) reveals that about 24.4 million Ghanaians use mobile phone [12]. The number of Internet users in Ghana in 2009 was 1.3 million, 93rd in the world, and reached 4.2 million in 2012, 69th in the world [13]. Also, NCA's mobile voice and mobile data market share trends for December 2015 reported that the number of mobile data subscribers in Ghana rose from about 17.73 million to 18.03 million, representing an access rate of 65.74 percent [14]. By the second quarter of 2016, mobile data subscription in Ghana according to the National Communications Authority had reached 18.8 million [15] This means that smart phone users in the country can have access to the Internet, the only other requirement being the availability of data. With the consequent exposure to ICT through the educational system, the potential for use of the Internet as a health seeking channel both is wide and should therefore be taken advantage of.

## 3. Justifying the Need for the Research

In spite of the evident infiltration of ICT accompanied by use of the Internet in sub-Saharan Africa, especially among the youth, not many studies have explored the use of Internet for health purposes. Studies in this area are largely concentrated on the developed world [6, 8, 9, 16–19]. There is a scarcity of research in Ghana done in this domain. The few works on Ghana have narrowly focused on teens in the junior high schools and other out-of-school youths [20–22]. It is thus not clear how university students, an important and recognizable segment of the population, with necessarily wide access to modern information technologies (mobile phones, desktop computers, laptops, and tablets) use the Internet for seeking health information and support.

Therefore, the aim of this study is to investigate the use of Internet among university students in Ghana for seeking online health information and support. The study specifically focuses on (1) access and use of Internet by students and (2) students' use of Internet for health purposes.

## 4. Methods

### 4.1. Study Design and Sampling

The study used a quantitative cross-sectional design and concentrated on 3 Ghanaian universities including 2 public universities (University of Ghana, Accra, and Kwame Nkrumah University of Science and Technology, Kumasi) and 1 private one (Central University, Accra). The study purposively focused on recruiting students at all levels (undergraduate, master's, and doctoral).

Convenient sampling was used to select a total of 740 students from the three universities. Of the 740 originally sampled university students, 650 participated by responding to the questionnaire, giving a response rate of 87.7%. These consisted of 250 each from University of Ghana and Kwame Nkrumah University of Science and Technology and 150 from the Central University. By the study protocol, only students from departments with inadequate health knowledge and information were included in the study. Students from departments more exposed to programmes or modules with adequate health-related information such as medicine, community health, nursing, public health, optometry, pharmacy, allied health, and other related faculties were not included. The sample also included undergraduate, master's, and doctoral students. Participation in the study was voluntary.

### 4.2. Research Instrument

Data were collected using the questionnaire. The researchers designed the questionnaire based on themes identified during the extensive review of literature. The questionnaire contained a mixture of different items ranging from multiple-choice to Likert-type scales and in some cases provided respondents with the opportunity for free expression.

The questionnaire consisted of 45 items grouped under 8 different headings labeled from A to H. Section A comprised 6 items and elicited demographic and background information of respondents including age, gender, level/year of study, type of accommodation, subject of study, and type of institution (whether public or private). The next 9 questions in Section B sought information on students' access and use of the Internet including questions on whether or not they use Internet, number of hours of Internet use per day, means of Internet access, type of Internet used on campus, and place of primary Internet access. Students were also asked to indicate their level of experience in using the Internet and also the challenges that they face in their access and use of the Internet.

Section C focused on the students' use of Internet for health purpose and covered issues like whether or not students have used the Internet for health purpose. Section D focused on how participants used the Internet, including frequency for specific health purposes. Frequency of use of Internet for specific a health purpose was measured using a 4-point Likert scale as follows: 1, always; 2, often; 3, occasionally; 4, never. The last item under this section was about the frequency of finding the health information sought by students using the Internet, and this was measured using a 5-point Likert scale as follows: 1, always; 2, most of the time; 3, only sometimes; 4, hardly ever; 5, never.

Similarly, Section E was on students' use of devices, apps, and platforms to obtain health information. Students were required to respond with Yes or No to their use or nonuse of selected devices, apps, and platforms. Section F focused on use of health information obtained by respondents using the Internet. Here too, students were required to respond with Yes or No to selected use of health information obtained. Under Section G, participants were required to rate the importance of identified factors affecting their decision to use apps, platforms, and websites as sources of health information. Specifically, participants were to indicate whether the selected factors were not important, fairly important, or very important. Section H, the last section, focused on how the health information obtained using the Internet had helped improve students' personal health.

### 4.3. Validity and Reliability of the Questionnaire

The researchers took measures to ensure validity of the developed questionnaires. Before the actual data collection, the questionnaire was reviewed by 2 academics including one statistician and one medical geographer. Also, 5 postgraduate students including 3 from Kwame Nkrumah University of Science and Technology and 2 from the University of Ghana reviewed the questionnaire for content validity. Modifications were made based on the feedback from the reviewers. With the aim of improving its reliability, the questionnaire was then pretested on 15 university students who were not part of the actual survey. Modifications were also made based on the results of the pretesting. Internal consistency reliability was ensured by calculating Cronbach's alpha coefficient of internal consistency. A value of .697 was obtained as Cronbach's alpha coefficient, indicating acceptable level of internal consistency in the questionnaire.

### 4.4. Data Collection

The questionnaires were administered by the researchers with the help of three trained graduate students. The questionnaires were sent to the assistants from the 3 selected universities via email to be printed for administration. The assistants were first taken through the questionnaire to ensure familiarity and ease of handling. Participation in the study was voluntary, and informed consent was obtained before the questionnaire administration began. To ensure consistency, the questionnaire was designed and printed in the English language, which is the official language of instruction in Ghanaian academic institutions.

### 4.5. Data Analysis

Data were analyzed using frequencies and percentages, as well as means and standard deviations. Descriptive statistics were calculated for the following: sociodemographics, access and general use of the Internet, use of Internet for health purpose, usage of health information sought, and rating of the importance of criteria for evaluating health websites, apps, and platforms, as well as the importance of health information sought in improving students' health. Data analysis was done using the IBM SPSS Statistics version 20.

## 5. Results

### 5.1. Characteristics of the Sample

Table 1 presents the background characteristics of the study participants. Variables of interest are age, gender, level of study, and place of residence. Participating in the study were 650 students from three (3) Ghanaian universities. The participants in the study were aged between 18 and 37 years, with an average age of 27 years. For the age groups, the majority (35.5%) belonged to the 20–24 category, followed by those aged 25–29 years (23.8%).

The majority of the respondents were males (*n* = 383, 58.9%). By level of study, undergraduate students formed the majority (*n* = 409, 62.9%), followed by master's students (*n* = 186, 28.8%), with only a few doctoral students (*n* = 55, 8.5%). The students either lived on campus in the traditional halls of residence (*n* = 234, 36.0%), or in private hostels (*n* = 30.3%), or stayed with their families at varying distances from the main campus (206, 31.7%).

### 5.2. Students' Access and Use of Internet

Table 2 presents results on students' access to Internet services. All of the 650 participants confirmed that they access and use the Internet, and a great majority (*n* = 509, 78.3%) reported Internet use every day. Also, most students had used the Internet for more than five years, with as many as 48.5% (*n* = 315) having used the Internet between 5 and 9 years. Another 28.6% (*n* = 186) indicated Internet use for a continuous period of not less than 10 years. Per duration of interne use a day, most students use the Internet for up to seven hours a day, and specifically 37.5% (*n* = 244) use Internet for a maximum of three hours a day, followed by usage of between 4 to 7 hours a day by 33.4% (217) of students. Few students (*n* = 60, 9.2%) use the Internet for more than 10 hours daily.

The students mostly accessed internets using mobile data bundles (*n* = 268, 41.2%) and campus WiFi (*n* = 195, 30.0%). Few students (*n* = 33, 5.1%) use Local Area Network (LAN). On the place of primary Internet access, students access Internet services mostly on campus (WiFi and labs) (*n* = 294, 45.2%) and halls and hostel (*n* = 229, 35.2%). Only a few accessed Internet from their homes (*n* = 107, 16.5%). The students' experience in using Internet was acceptable, as the majority of them rated their level of experience in using Internet as fair (*n* = 364, 56.0%), followed by those who rated themselves as very experienced (*n* = 251, 38.6%). Few students rated themselves as not experienced (*n* = 22, 3.4%).

The students were asked to indicate the main challenge that they face in accessing and using Internet. More than half (*n* = 351, 54.0%) indicated unreliable and slow connection as their main challenge. Other challenges reported on were high cost of Internet and devices, reported by 14.9% (*n* = 97), problems of viruses and malware (7.8%), and congestion at ICT centres where Internet is accessed (6.0%). Unreliable power connection was surprisingly not an issue, reported by only 5.5% of the sampled students.

### 5.3. Students' Use of the Internet for Health Purpose

The students were asked about their extent of use of Internet for health purposes. They were first asked whether or not they used the Internet for health purposes or to seek health information. The results are presented in Table 3 which also presents results on the reasons for their use of Internet for health purposes. Out of the 650 students, the overwhelming majority 67.7% (*n* = 440) use Internet for health purposes or to seek health information.

The study also enquired about factors that students take into consideration in their selection of the means of accessing the Internet for health information and support. As shown in Table 4, currency of information was the most important factor, followed by ease of understanding of information. The least important factors were quality of links and comprehensiveness.

The students use the online health information to take several important decisions and actions concerning their health. As shown in Table 5, greater majority of the students reported that they mostly use the health information obtained as a basis for lifestyle change (*n* = 315, 72.4%). There is a big lag between this response and the next important use of online health information, which is “discussing health issues with health professionals” (*n* = 170, 39.5%). Other self-reported uses of online health information are “changing medication” (*n* = 102, 23.7%), and making, cancelling, or changing appointments with doctor (*n* = 63, 14.7%).

The survey further revealed the extent of self-reported improvement in students' health conditions by using information sought using the Internet. As shown in Table 6 majority (*n* = 144, 40.1%) revealed that their health conditions have “improved a lot,” followed by those whose health has “improved somehow” (*n* = 112, 33.5%). Altogether, these two responses show a positive impact of online health information on respondents' health as they make up 73.6% of responses.

Only a small 1.2% (*n* = 4) of students reported that online health information had not had any effect at all on their overall health situation.

## 6. Discussion

This study provides insight into the extent of Internet use for online health information and support among university students in Ghana. The study revealed that all the university students use the Internet. This finding is not surprising, looking at the rising popularity of the Internet among the youth and the ease of access by android, IOS, and Microsoft-powered smart phones and other hand-held devices. Earlier studies have found an extensive use of Internet among adolescents and young adults including university students [5, 20, 21, 23–26]. Most tertiary institutions in Ghana currently provide Internet connections for their students whether in their halls and hostels or on campus by means of campus-wide WiFi and computer laboratories. Respondents in this study primarily accessed the Internet on campus as well as in the halls and hostels, possibly because such places mostly offer reliable Internet services at low costs. They are also places where students undertake academic and research activities whether in their busy or leisure periods. This finding is consistent with previous studies [5, 11, 21, 24, 25, 27–29].

The survey revealed that more than half of all participants use the Internet to obtain health information and support (among other uses), specifically finding information about illnesses and interacting with health professionals via emails, Facebook pages, and other social media platforms such as Whatsapp, where contact details of selected health professionals at the university hospitals have been made available. This implies that there is a great potential of the Internet as an important channel for health information and support, for not only university students, but also the general population. This is particularly so as the Internet offers suitable, a lot of, and cheap information as noted by Horgan and Sweeney [6]. Also, the Internet is seen as the acceptable and practicable tool for behavioural intervention for university students as noted by Zabinski et al. [30] and Escoffery et al. [31] and adults in general as noted by Tate et al. [32].

The survey also revealed that about 68% of the sampled university students in Ghana sought for health information or support online. This result compares with other studies globally, including a study on predictors of online health seeking behaviour among Egyptian adults which shows that Internet was the main source for health information [33]. It also confirms the result of a survey in 7 European countries (Denmark, Norway, Germany, Poland, Greece, Portugal, and Latvia) in 2005 which revealed that, on average, about 63% of individuals aged 8–29 years were online health seekers [34]. Also, the result of a survey in Italy in 2010 showed that 65% and 60% of young Italian females and males (between 18–29 years) respectfully used the Internet for health-related purposes [35]. It also confirms a study in the United States on health information seeking behaviour among US adults using data from four cycles (2011–2014) of the Health Information National Trends Survey (HINTS) that found that a greater percentage of US adults use the Internet as the first place they go for health information [36]. A systematic literature review on the use of social media for retrieving health information by patients and healthcare consumers revealed that there is a high use of social media by patients and healthcare consumers in retrieving health-related information [37]. Several other studies in the United States of America [31, 32], Italy [35], and Israel [38] have also reported lower levels of Internet use for health purposes than those found in the present study. Reasons for the higher use of the Internet for health purposes by the university students could be the abundance of Internet access on campus including campus-wide WiFi and computer pools and the comfortability with which students find their use [4, 38]. There is little wonder then that more than half of the students reported being experienced with the Internet. The level of Internet use for health purpose in this study was however lower than level reported in Australia [39] where two-thirds of the participants use Internet for health purposes. Also, in the United States, the Pew Research Center's Internet & American Life Project in 2012 showed that 72% of people aged 18–29 years were online health seekers [40].

The wealth of health websites and online information available to the public has raised numerous concerns about how accurate the information on such websites and platforms is as well as the possibility of harm as a result of inaccurate information [4, 41]. Criteria for assessing which websites and platforms to use to gain access to health information varied from accuracy, currency of information, comprehensiveness, ease of understanding, readability, confidentiality, interactivity, and quality of links to use of multimedia and appearances. Barnes et al. [42] found that the important criteria among adult Internet users included authority of the sources, ease of use, attribution, disclosure of authors, accessibility, and availability. This study consequently confirms that the credibility of the websites, platforms, and information is critical for consumers of online health information. However, the students may face some difficulties with regard to quality and accuracy of health-related information obtained. It is therefore essential to help them search and use the most suitable and quality online health information. This can be achieved by targeting the main way of Internet use, which is identified to be the social networks. Social networks could be established as a place for health professionals to help the youth deal with online information as noted by Buame [20].

Finally, 72% of the students who use Internet for health purpose reported having had their lifestyle or health behaviour change after their contact with online information. It is possible that the changes in question reflect improved healthy lifestyles (e.g., good eating habit, adequate exercise, reducing alcohol intake, and smoking).

## 7. Conclusions

This study reveals that Ghanaian university students are active users of the Internet, including use for health purpose (i.e., searching for health information and support). The results justify the increase in efforts over the past several years by the various universities in Ghana to provide cheap and accessible Internet for students for various purposes, most of which are development-oriented and life-enhancing.

This study concludes that the Internet is assuming a more and more important role in the lives of the youth especially students in higher education institutions and that its use is not only limited to purely academic and leisure purposes, but also used for life-supporting and sustaining purposes including searching for health information. There is therefore the need to promote e-health in the country as a viable platform for health interventions and healthcare. This would, among other benefits, provide quick, accessible, and affordable access to healthcare and health information. It would also ease the pressure and congestion that characterize physical healthcare services in the country, leading to overall improvement in healthcare delivery. For the youth and especially tertiary students, providing on-the-go access to healthcare via e-health fits into their lifestyle and is therefore very appropriate. In this vein, it is expected that, as per results of this study, the Ghana Health Service and its supervising Ministry of Health will take the giant steps of making healthcare truly accessible to all as enshrined in international and national protocols by promoting e-health through design of responsive websites, platforms, and applications for accessing healthcare.

It is envisaged that the integration of technology in healthcare, by promoting Internet-based access to healthcare especially with university students and other qualified groups in the population, will go a long way to address problems of access and quality in healthcare delivery in the country. However, it would be equally prudent to be aware of online safety issues for students and other users of Internet for health-based information. Piracy issues will also have to be addressed, together with providing information on privacy options.

There are limitations to the work. The study was conducted among university students and thus cannot lay claim to be representative of the behaviour of all young people in the country. The study also assumes that Internet-based health information is wholesome and appropriate to the needs of seekers, which might not necessarily be so. There is junk information out there, and the extent to which this might impact health outcomes must be investigated and safeguarded. In pursuit of this, there is the need for a thorough evaluation of the various websites, apps, and platforms that are health related, so that those that are not health-enhancing could be avoided.

## Supplementary Material

Supplementary Material-Questionnaire: The questionnaire consisting of 25 main items was used to obtain data from the sampled students from the universities on their health information seeking behaviour using the internet.

## Figures and Tables

**Table 1:** Background characteristics of respondents.

Variable	Category	Frequency (*n* = 650)	*Percentage*
Age (years)	<20	139	*21.4*
	20–24	231	*35.5*
	25–29	155	*23.8*
	≥30	115	*17.7*
	Missing	10	*1.5*
	**Mean**	**27.40**	
	**SD**	**3.47**	

Gender	Male	383	*58.9*
	Female	267	*41.1*

Level of study	Undergraduate	409	*62.9*
	Master's	186	*28.6*
	Doctoral	55	*8.5*

Place of residence	Campus	234	*36.0*
	Hostel	197	*30.3*
	Home	206	*31.7*
	Missing	13	*2.0*

**Table 2:** Internet access and use by students.

Variable	Categories	Frequency (*n* = 650)	*Percent*
Use of Internet	Yes	645	100.0
	No	0	0.0

Years of Internet use	<1	8	*1.2*
	1–4	126	*19.4*
	5–9	315	*48.5*
	≥10	186	*28.6*
	Missing	16	*2.3*

Use of Internet everyday	Yes	509	*78.3*
	No	136	*20.9*
	Missing	5	*.8*

Hours of daily use Internet	1–3	244	*37.5*
	4–7	217	*33.4*
	8–10	86	*13.2*
	>10	60	*9.2*
	Missing	43	*6.6*
	**Mean**	**5.41**	
	**SD**	**3.28**	

Type of Internet	Mobile data	268	*41.2*
	Campus WiFi	195	*30.0*
	LAN	33	*5.1*
	All	149	*22.2*
	Missing	5	*.8*

Place of primary Internet access	Campus labs and WiFi	294	*45.2*
	Halls and hostels	229	*35.2*
	Home	107	*16.5*
	Missing	20	*3.1*

Level of experience	Very experienced	251	*38.6*
	Fairly experienced	364	*56.0*
	Not experienced	22	*3.4*
	Missing	13	*2.0*

Barriers to Internet use	Unreliable and slow connection	351	*54.0*
	High cost of internet and devices	97	*14.9*
	Congestion at ICT centres	39	*6.0*
	Unreliable power supply	36	*5.5*
	Viruses and malware	51	*7.8*
	No challenge	71	*10.9*
	Missing	5	*.8*

**Table 3:** Students' use of Internet for health purpose.

Response	*n*	%
*Use of Internet for health purpose*		
Yes	440	67.7
No	205	31.5
Missing	4	.8
Total	650	100.0
*Reasons for using Internet for health purpose*		
Vast amount of valuable information available	249	56.6
Anonymous, private, and confidential	340	77.3
Easy to find information	421	95.7
Cheap	170	38.6
Convenient	310	70.5
Easy to communicate with peers	280	63.6
Less embarrassing than talking to a professional	216	49.1

**Table 4:** Factors considered in seeking online health information.

Factor	Ratings			Total
	Not important	Fairly important	Very important	
	*n* (%)	*n* (%)	*n* (%)	
Accuracy of information	1 *(1.4)*	175 (40.9)	247 (57.7)	428
Currency of information	16 (3.7)	184 (43.0)	288 (67.3)	428
Comprehensiveness	11 (2.6)	215 (50.0)	204 (47.4)	430
Ease of understanding	10 (2.3)	156 (36.0)	267 (61.7)	433
Content readability	3 (.7)	178 (41.6)	247 (57.7)	428
Confidentiality	63 (14.5)	204 (47.1)	166 (38.3)	433
Interactivity	74 (17.1)	233 (53.8)	126 (29.1)	433
Quality of links	33 (7.6)	220 (50.8)	180 (41.6)	433
Use of multimedia	68 (20.6)	164 (49.7)	98 (29.7)	330
Appearances	36 (10.9)	174 (52.7)	120 (38.4)	330

**Table 5:** Decisions students make with online health information.

Use of online health information	Response				Total
	Yes		No		
	Freq.	%	Freq.	%	
Making, canceling, or changing appointment with doctor	63	*14.7*	367	*85.3*	430
Discussing health issues with health professional	170	*39.5*	260	*60.5*	430
Changing medication, without discussing with professional	102	*23.7*	328	*76.3*	430
Change of lifestyle	315	*72.4*	120	*27.6*	435

**Table 6:** Extent of improvement in students' health by online health information.

Extent of improvement	Frequency	Percentage
A lot	134	40.1
Somehow	112	33.5
Only a little	84	25.1
Not at all	4	1.2
Total	334	100.0
Missing	106	
