# Supplementary material for: The Internet Use for Health Information Seeking among Ghanaian University Students: A Cross-Sectional Study
Source: Int J Telemed Appl. 2017 Oct 31;2017:1756473. doi: 10.1155/2017/1756473 (PMC5684546; doi:10.1155/2017/1756473)
Supplement: Supplementary file 1 — Supplementary Material-Questionnaire: The questionnaire consisting of 25 main items was used to obtain data from the sampled students from the universities on their health information seeking behaviour using the internet. [file 1756473.f1.pdf]

QUESTIONNAIRE

The aim of this study is to assess the use of the internet for health purposes among students in Ghanaian Higher Education Institutions. You are therefore required to answer the questionnaires below, as best as you can. Thank you!

Demographic Data

1. Age group: (1) < 20 years [ ] (2) 20-24 years [ ] (3) 25-29 years [ ] (4) 30-34 years [ ] (5) 35 and above [ ]

2. Gender (1) Male [ ] (2) Female [ ]

3. Level/Year (1) 100 [ ] (2) 200 [ ] (3) 300 [ ] (4) 400 [ ] (5) Masters [ ] (6) Doctoral [ ]

4. Program of study..

5. Place of Attendance while attending school (1) Campus [ ] (2) Hostel [ ] (3) Home [ ]

6. Type of University (1) Public [ ] (2) Private [ ]

Access and Use Internet

6. Do you use the internet? (1) Yes [ ] (2) No [ ]

7. If YES, indicate how long have you used internet (in years) ………………………………….

8. Do you use the internet every day? (1) Yes [ ] (2) No [ ]

9. Indicate the number of hours you use internet per day …………………………………………

10. By what means do you access the internet? (1) Smartphone [ ] (2) Laptop [ ] (3) Tablet [ ] (4) Desktop computer [ ]

9. What type of internet do you use on campus (1) Mobile Data [ ] (2) Campus Wifi [ ] (3) Both [ ] (4) Other (specify) …………………………………………………

10. Indicate your place of primary internet access (1) Campus Labs and Wifi [ ] (2) Halls and Hostels [ ] (3) Home [ ] (4) Other (specify)……………………………..

11. Indicate your level of experience with the internet (1) Very Experience [ ] (2) Fairly experience [ ] (3) Not experienced [ ]

12. What barriers do you encounter in your use of internet? ………
………
………
………
………
……..

Use of Internet for Health Purpose

13. Do you use the internet to get health information/ for health purpose? (1) Yes [ ] (2) No [ ]

14. If YES, how often do you use internet for the following health purposes?

i. Find information that can help you decide whether to consult a health professional (1) Always [ ] (2) Often [ ] (3) Occasionally [ ] (4) Never [ ]
ii. Find health information prior to an appointment (1) Always [ ] (2) Often [ ] (3) Occasionally [ ] (4) Never [ ]
iii. Find information after an appointment with a health professional (1) Always [ ] (2) Often [ ] (3) Occasionally [ ] (4) Never [ ]
iv. Interact with health professionals you have met face to face (1) Always [ ] (2) Often [ ] (3) Sometimes [ ] (4) Never [ ]
v. Participate in forums or self-groups (focusing on health or illness) (1) Always [ ] (2) Often [ ] (3) occasionally [ ] (4) Never [ ]
vi. Read about health and illness including symptoms and causes (1) Always [ ] (2) Often [ ] (3) occasionally [ ] (4) Never [ ]

15. What is the frequency of finding the health information that you look for using the internet? (1) Always [ ] (2) most of the time [ ] (3) only sometimes [ ] (4) hardly ever [ ], (5) never [ ]

Students' Usage of Devices, Apps and Platforms

14. Have you used any of the following as a source of health information?

i. Apps for smart phones and pad (1) Yes [ ] (2) No [ ]
ii. Search engines such as Google, Bing and Yahoo (1) Yes [ ] (2) No [ ]
iii. Social media such as Facebook and Twitter (1) Yes [ ] (2) No [ ]
iv. Video services such as YouTube (1) Yes [ ] (2) No [ ]

v. Emails (1) Yes [ ] (2) No [ ]

vi. Websites (1) Yes [ ] (2) No [ ]

Students' Usage of Information sought on the Internet

15. Did you do any of the following after seeking health information on the internet?

i. Make, cancel or change appointment with your doctor (1)Yes [ ] (2) No [ ]

ii. Discuss the health information obtained with a doctor or health professional (1) Yes [ ] (2) No [ ]

iii. Change your medication if any, without discussing it with your doctor? (1) Yes [ ] (2) No [ ]

iv. Change your lifestyle? (1) Yes [ ] (2) No [ ]

Students' rating of Importance of Health Information Sought using the Internet

If you have read a website or used a platform with health information, rate the importance of each of the following factors to you.

Number	Factor	Not Important	Fairly Important	Very Important
16	Accuracy			
17	Currency of Information			
18	Comprehensiveness			
19	Ease of Understanding			
20	Readability			
21	Confidentiality			
22	Interactivity			
23	Quality of Links			
24	Use of Multimedia			
25	Appearances			

Attitudes and Beliefs about the use of internet for finding health information

26. Indicate the frequency of finding health information that you look for (a) Always [ ] (b) Most of the time [ ] (c) Only sometimes [ ] (d) Hardly ever [ ] (e) Never [ ]

27. Indicate how obtaining health information on the Internet has improved your personal health. (a) A lot [ ] (b) Some [ ] (c) Only a little [ ] (d) Not at all [ ]
